# New Frontiers in Wine Microbiology

**DOI:** 10.3390/foods10051077

**Published:** 2021-05-13

**Authors:** Aspasia Nisiotou

**Affiliations:** Hellenic Agricultural Organisation “DEMETER”, Sofokli Venizelou 1, GR-14123 Lykovryssi Attikis, Greece; anisiotou.wi@nagref.gr

The wine sectoris currently facing new challenges. The global climatic change is a primary factor that severely affects viticulture and winegrowing, posing a threat for the sustainability of the grape/wine sector in several regions [1]. Health issues raised by consumers also redirect the priorities of the sector. Last but not least, the increasing competition in the world wine market set severe pressures for the industry. In this context, genetic microbial resources in vineyard ecosystems and novel biotechnological applications may benefit winemaking in various ways to face current challenges.

In the current Special Issue, one review [2] and eight original research papers [3,4,5,6,7,8,9,10] were released. An outline of these publications is presented here. Four papers [4,5,6,10] focused on the exploration of the genotypic and phenotypic diversity of the indigenous yeast and bacteria of the grape/wine system and their potential exploitation in winemaking. In the study of Chalvatzi et al. [4], the spatiotemporal genetic diversity of *Saccharomyces cerevisiae* associated with vineyards of Santorini, a small Aegean island was assessed. Despite the small spatial scale, the genetic diversity of *S. cerevisiae* was relatively high. Significant differences in the populations were observed among vineyards and between vintages. The authors also studied the invasion of commercial starters (rather, escapees from wineries), which may constitute a potential risk in terms of loss of the local yeast biodiversity. Interestingly, the industrial strains do not dominate over the natural strains, and their high abundance seems to be temporary.

Mateus et al. [6] provided insights on the non-*Saccharomyces* (NS) fraction of the yeast microbiota naturally present in Port wine fermentations for the first time. NS yeasts are of particular importance in Port wine production, in which the fermentation is early terminated. The authors identified *Hanseniaspora uvarum*, *Lachancea thermotolerans*, and *Metschnikowia pulcherrima* as the most abundant NS yeasts. Notably, the respective populations also exhibited particularly high genotypic and phenotypic biodiversity when subjected to the stress conditions encountered in winemaking environment. 

Native lactic acid bacteria (LAB) have emerged as an attractive alternative to commercial formulations for conducting the malolactic fermentation, since the latter are not always successfully implanted in different wines. Therefore, there is an increasing interest in the selection of autochthonous strains that appear better adapted to specific wine types in different wine-growing regions. López–Seijas et al. [5] evaluated malolactic bacteria isolated from wines of Albariño grape cultivar for use in winemaking. The authors identified six species (which mostly belong to *Lactobacillus*) that exhibited biogeographical distribution. Five strains with malolactic activities that did not produce biogenic amines or volatile phenols were identified as potential starters. To counteract the difficulties in conducting malolactic fermentation under stress conditions, Pannella et al. [9] investigated the potential of the biofilm cells of *Lactobacillus plantarum*. The authors studied the biofilm formation capacity under stress conditions in different *L. plantarum* strains to select the best performing strain. By using oak supports for biofilm formation, it was shown that the persistence in biofilm cells was higher than in planktonic cells, ensuring a fast and complete conversion of L-malic acid.

In view of the global climate change, the use of selected NS yeasts may counteract the resultant adverse effects on wine quality, such as the reduced acidity and the increasing ethanol content. Sgouros et al. [10] considered the potential of a native high lactate-producing *L. thermotolerans* strain (P-HO1) for use in winemaking. The biological acidification is an attractive alternative to counteract high pH values in wines, which are likely to increase due to global warming. P-HO1 produced the highest levels of lactic acid (10.4 g/L) ever recorded in mixed fermentations and lowered ethanol by 1.6 % vol. Following comparative transcriptional analysis of the lactate dehydrogenase (LDH) genes (which encode the key enzyme for lactate biosynthesis), along with the alcohol dehydrogenase paralogs (ADHs), the authors concluded that LDH2, but not the other LDHs or ADHs, may be involved in the elevated production of lactic acid at the cost of ethanol. An interesting alternative to achieve the production of low ethanol wines was proposed by Nikolaou et al. [7], who applied an immobilized kefir culture on natural supports. High values of ethanol productivity and malic acid conversion were recorded, which allowed the simultaneous completion of alcoholic and malolactic fermentations.

Keeping human health in mind, two studies evaluated strategies to reduce sulphur dioxide (SO_2_) levels in wines. In the original research of Pachnowska et al. [8], the use of silica nanoparticles (SiO_2_) was proposed as a healthier alternative to SO_2_ for microbiological stabilization. Silica nanospheres were shown to cause a disruption of *Oenococcus oeni* cells, leading to population reduction in young wines when treated with SiO_2_. The need to restrict sulphide and sulphite levels in organic and sulphite-free wines necessitates the use of strains that produce low amounts of sulphur compounds. Agarbati et al. [3] selected three new strains through sexual mass-mating spores’ recombination of a native *S. cerevisiae* isolate, which exhibit low sulphide and sulphite production and good volatile profile. These strains may serve as starters in the production of ‘clean wines’, as extensively reviewed by Maykish et al. [2]. The fast growing organic wine industry has caused a prompt interest in the evolution of biodynamic, natural, and clean wine. Clean wine, in particular, may allow for wine consumption by consumers who otherwise suffer from negative effects related to sulphite levels and histamines in wines.

The contributions gathered in this Special Issue highlight the new frontiers in wine microbiology research in an effort to address current concerns in winemaking. Novel and selected special yeast and bacterial strains provide promising alternatives to both established and new challenges in wine fermentations. Novel biotechnological strategies may be applied to treat health and product stabilization issues. We hope that these findings provide some means to efficiently address some of the emerging challenges in the field.
